# Positive Emotional Language in the Final Words Spoken Directly Before Execution

**DOI:** 10.3389/fpsyg.2015.01985

**Published:** 2016-01-13

**Authors:** Sarah Hirschmüller, Boris Egloff

**Affiliations:** Department of Psychology, Johannes Gutenberg University MainzMainz, Germany

**Keywords:** mortality salience, language use, quantitative text analysis, emotional positivity, terror management

## Abstract

How do individuals emotionally cope with the imminent real-world salience of mortality? DeWall and Baumeister as well as Kashdan and colleagues previously provided support that an increased use of positive emotion words serves as a way to protect and defend against mortality salience of one’s own contemplated death. Although these studies provide important insights into the psychological dynamics of mortality salience, it remains an open question how individuals cope with the immense threat of mortality prior to their imminent actual death. In the present research, we therefore analyzed positivity in the final words spoken immediately before execution by 407 death row inmates in Texas. By using computerized quantitative text analysis as an objective measure of emotional language use, our results showed that the final words contained a significantly higher proportion of positive than negative emotion words. This emotional positivity was significantly higher than (a) positive emotion word usage base rates in spoken and written materials and (b) positive emotional language use with regard to contemplated death and attempted or actual suicide. Additional analyses showed that emotional positivity in final statements was associated with a greater frequency of language use that was indicative of self-references, social orientation, and present-oriented time focus as well as with fewer instances of cognitive-processing, past-oriented, and death-related word use. Taken together, our findings offer new insights into how individuals cope with the imminent real-world salience of mortality.

## Introduction

Final words written or spoken shortly before death have fascinated people and have been collected in writing for a long time (e.g., Marvin, 1901; Brahms, 2010). Anything but banal word use when confronting death can offer researchers valuable insights into how people cope with existential threats and the human psyche in general (Pennebaker et al., 2003). Hence, final words in the form of rehearsed or impromptu sayings spoken by a dying person can reveal how individuals emotionally regulate the salience of imminent mortality. Here, we analyzed emotional language use in the final statements of executed Texas death row inmates and compared our findings with rates of positive emotion word use in general and in contexts involving contemplated death and attempted or actual death by suicide.

Intuitively, one might imagine that thoughts of one’s own death should evoke fear and anxiety as death may be associated with a broad range of frightening aspects (i.e., pain, loss of loved ones, unfulfilled goals; e.g., Niemeyer and Moore, 1994; Florian and Mikulincer, 1997). Psychological denial of death to escape the anxious awareness of our mortality constitutes one of the most basic drives in individual behavior (Becker, 1973). According to Terror Management Theory (TMT; Greenberg et al., 1986), individuals employ a wide range of cognitive and behavioral efforts to regulate the anxiety that mortality salience evokes (Greenberg et al., 1997; Pyszczynski et al., 2004). These psychological defense mechanisms are aimed at maintaining self-esteem and acquiring meaning in life (Pyszczynski et al., 1999).

Empirical findings by DeWall and Baumeister (2007) suggest that an automatic orienting toward emotionally positive information and associations serves as a way to protect and defend against mortality salience. In a series of studies, the authors showed that thinking about death (vs. thinking about dental pain) activated a non-conscious emotional coping response that was counterintuitive to the overt emotional distress one might expect, such that participants completed ambiguous word stems with relatively more positive emotion words and favored positive emotional associations in judgments of word similarity. Also, findings by Kashdan et al. (2014) indicated that such a shift toward the use of positive emotion words may be involved in regulating the fear of death: writings of individuals contemplating death (vs. people thinking about dental pain, uncertainty, or meaninglessness) showed an increased use of positive emotional language.

Although these studies offer important insights into the psychological dynamics of mortality salience, they are limited by the fact that the situation created by letting university students contemplate death in a standardized lab situation necessarily differs in several aspects from real life and real death. Consequently, in an attempt to better understand the feelings of individuals prior to their imminent actual death, researchers have conducted content analyses of suicide notes (Tuckman et al., 1959; Handelman and Lester, 2007) and other writings left behind after suicides (e.g., diaries, poetry; Stirman and Pennebaker, 2001; Pennebaker and Stone, 2004). Tuckman et al. (1959) reported that the emotional content of 165 analyzed suicide notes was surprisingly positive and contained expressions of gratitude and affection. Similarly, research has shown that notes from completed suicides contained more positive emotional language than notes from attempted suicides (Handelman and Lester, 2007) and that writings that occurred temporally closer to death were higher in positive emotional language use (Pennebaker and Stone, 2004).

Without any doubt, the psychological “terror” felt in the situation of self-decided death by suicide is extreme. However, there may be one situation where individuals face an even greater amount of terror: directly before death by execution. This situation is characterized by a complete absence of controllability and a maximal subjection to powerful others who have the right to end one’s life. Consequently, last words as part of the execution process, visible as early as 1388 in England (Howell, 1809), provide a unique opportunity for exploring the predictions of TMT.

An additional theoretical perspective that can help shed light on emotional regulation as reflected in final statements before execution is provided by socioemotional selectivity theory (SST; Carstensen et al., 1999). According to SST, the perception that one has limited future time (e.g., caused by a temporal proximity to the end of one’s life) is postulated to increase individuals’ motivation to derive emotional meaning by prioritizing close social relationships over instrumental or knowledge-related goals (e.g., Carstensen et al., 1999; Carstensen, 2006). Research has provided evidence that individuals who perceive their future time as limited or who have limited actual life expectancies strongly value emotionally close social partners (e.g., Carstensen and Fredrickson, 1998; Fung et al., 1999, 2001; Fung and Carstensen, 2006). Moreover, as individuals recognize the inevitable constraints on time that are imposed by one’s mortality, which is not necessarily associated with chronological age for death row inmates, the focus on the present moment is likely to increase (Carstensen et al., 1999).

In the present research, we analyzed the actual final statements made by executed death row inmates in the state of Texas. This unique data set provided the opportunity to analyze how people cope with immediate death in a standardized, uncontrollable situation in a large sample. Prior research found some indications of positivity by using qualitative content coding, implemented by humans, of the prevalent thematic categories found in Texas death row inmates’ last statements, the most prominent themes being the expression of love or appreciation (Heflick, 2005; Schuck and Ward, 2008). Further themes that were identified included addressing others; seeking forgiveness; expressing self-comfort, wishes, or hopes; and religious references (Schuck and Ward, 2008). In contrast to these previous studies, we used computerized quantitative text analysis (Mehl, 2006) as a more objective measure of emotional language use. In the following, we outline the important advantages of the methodology we applied and the relevant research questions that we were thus able to address.

Quantitative text analysis can be differentiated with regard to “what” (i.e., content) a person is saying and “how” (i.e., style) the person is saying it, whereby “how” the person says something can reveal more subtle aspects of communication (Mehl, 2006). Issues that occur with human coders consist of a hastiness to ascribe meaning and a general inability to monitor word choice (e.g., Hart, 2001). Thus, given these issues and the fact that different content themes (e.g., appreciation/love; expressing self-comfort or wishes) may be expressed through the same linguistic style (e.g., emotion-related words), a computerized word-count-based text analysis allows researchers to objectively and reliably assess (i.e., count) the linguistic features of individuals’ statements and detect subtle linguistic styles (Pennebaker and King, 1999; Pennebaker et al., 2003; Mehl, 2006; Tausczik and Pennebaker, 2010). Subsequently, the linguistic features that are assessed (e.g., percentage of positive emotion words in death row inmates’ final statements) can be (a) compared with word usage rates in samples contemplating death or experiencing a limited time horizon due to a decision to commit suicide, (b) related to the demographic characteristics of the sample of death row inmates (e.g., age at execution, years on death row, or educational level), and (c) associated with other linguistic markers (e.g., pronoun use, verb tense, word use indicative of psychological processes such as social orientation or cognitive processing).

We hypothesized that if tuning in to emotional positivity acts as a psychological mechanism aimed at coping with the threat of mortality (DeWall and Baumeister, 2007; Kashdan et al., 2014), it would be reflected in the emotional word use in death row inmates’ final statements such that death row inmates would use a higher proportion of positive than negative emotion words. We further hypothesized that the immense existential threat that real-life executions evoke would lead to a higher proportion of positive emotion words compared with word usage base rates (Pennebaker et al., 2007b) as well as compared with the words of individuals contemplating death (Kashdan et al., 2014) or individuals attempting or committing suicide (Handelman and Lester, 2007). Drawing on postulations by SST (Carstensen et al., 1999), we further aimed to explore the relations between positive emotional word use and language indicative of self-references, social orientation, cognitive processing, time orientation, and personal concerns with religion and death. We expected that emotional positivity in executed death row inmates’ final statements would be associated with a greater use of social-orientation words and present-tense verbs. Furthermore, we conducted an exploratory investigation of associations between positive emotional language use and self-references, cognitive-processing word use, past-tense and future-tense verb use, as well as religion and death-related word use.

## Materials and Methods

### Sample

To test our hypotheses, we analyzed death row inmates’ final statements, spoken minutes before their executions in the US state of Texas. The final statement transcripts as well as death row inmates’ demographic information were provided publicly available on the Texas Department of Criminal Justice (2015) website. Information about whether death row inmates abandoned their appeals and volunteered for execution was provided publicly available on the Death Penalty Information Center (2015b) website. As we only reanalyzed these publicly available data on a de-identified and anonymized aggregate level, a particular ethical approval for our study was not required. Of the 527 death row inmates executed between December 1982 and June 2015, transcriptions of spoken last statements were not available for 119 death row inmates. Of these 119 death row inmates, 108 were listed as having declined a last statement, eight had no statements provided, and three had only written statements provided. **Table 1** presents demographic information consisting of age at time of execution, age at incarceration, years spent on death row, and educational level (i.e., highest grade completed) for death row inmates with and without transcribed spoken final statements. Of the 408 death row inmates with transcribed spoken statements, one was excluded from analyses due to a reported higher age at incarceration compared to age at execution on the Texas Department of Criminal Justice (2015) website. The analyzed sample of death row inmates for whom spoken last statements were provided who were executed between 1982 and 2015 consisted of *N* = 407 (404 male, three female) individuals; 178 (43.7%) death row inmates were reported as White, 150 (36.9%) as Black, 77 (18.9%) as Hispanic, and two (0.5%) as another ethnicity. Death row inmates with no transcribed spoken last statement on record did not differ from the analyzed sample of 407 death row inmates in ethnic background, χ^2^(3) = 1.25, *p* = 0.740, Cramer’s *V* = 0.05, or on the other demographic variables, all *p*s > 0.09 (see **Table 1**).

**Table 1:** Demographic information on death row inmates executed between 1982 and 2015 (June 30) in Texas.

Demographic variables	Death row inmates with spoken statements					Death row inmates without spoken statements					Independent *t*-test			
				
	*n*	*M*	*SD*	Min	Max	*n*	*M*	*SD*	Min	Max	*df*	*t*	*p*	Cohen’s *d*
Age at execution	407	39.01	8.18	24.0	67.0	119	40.02	9.17	24.0	62.0	524	1.15	0.250	0.12
Age at incarceration	391	28.15	7.78	18.0	57.0	104	29.60	8.24	17.0	53.0	493	1.67	0.096	0.18
Years on death row	391	10.96	4.37	0	31.0	104	11.41	5.35	1.0	32.0	493	0.89	0.375	0.09
Education (highest grade completed)	381	10.11	2.11	0	16.0	100	10.39	2.04	5.0	16.0	479	1.20	0.231	0.14


*Demographic information of Texas death row inmates was obtained from the Texas Department of Criminal Justice (2015) website.*

### Data Preparation

Prior to the analyses, the transcript of the spoken final statement of each individual death row inmate was checked for additional remarks describing the inmate’s behavior and use of foreign language (e.g., “crying,” “portion of statement omitted due to profanity,” “speaking in French”). A cleaned text file was created by excluding additional remarks from the transcript and replacing foreign word use with English translations provided on the Texas Department of Criminal Justice (2015) website. Verbatim examples of Texas death row inmates’ last statements from the website are:

“I love my family. You all stay strong. Watch over each other. Stay strong. I love you. I love you. It’s my hour. It’s my hour. I love you. Stay strong.” (Executed death row inmate 360)

“Even though I lay on this gurney, seconds away from my death, I am at total peace. May the Lord Jesus Christ be with me. I am at peace. Hate is going on in this world and it has to stop. Hate causes a lifetime of pain. Even though I lay here I am still at peace. I am still a proud American, Texas loud, Texas proud. God bless America, God bless everyone. Let’s do this damn thing. Director Hazelwood, thank you very much. Thank you everyone. Spark, I love you, all of you. I love you Conna. It’s all good, it’s been a great honor. I feel it; I am going to sleep now. Goodnight, 1, 2 there it goes.” (Executed death row inmate 472)

“Yes, I do, uh at this time I would like to thank my parents who have been my pillar of strength throughout this. To my brothers and sisters and all my family members who have supported me and who have loved me despite my faults and imperfections. I would like to thank Pastor Williams for counseling me and guiding me. As I look to my right and I see the family of […]. I hope this brings you closure or some type of peace. I hope it helps his family, son and loved ones. This has been a long journey, one of enlightenment. It’s not the end, it’s only the beginning.” (Executed death row inmate 459)

### Data Analyses

The content of each transcribed last statement for each executed death row inmate was analyzed separately using a recent text analysis program, Linguistic Inquiry and Word Count (LIWC; Pennebaker et al., 2007a). LIWC uses an internal default dictionary and determines the percentage of words in a text or speech that correspond to various linguistic categories that have adequate psychometric properties (Pennebaker et al., 2007b). LIWC has been well-validated and has provided evidence for the psychological and social implications of word use (Pennebaker et al., 2003).

Only the output variables relevant to our hypotheses were examined in the current study. Specifically, we analyzed the following LIWC variables: total number of words, percentage of categorized dictionary words, percentage of positive emotion words, and percentage of negative emotion words. Examples of positive emotion words are *happy* and *love*; examples of negative emotion words are *sad* and *hate*. To explore associations between positive emotion word use and linguistic variables indicative of self-references and social orientation, we further analyzed the LIWC variable of the first-person singular pronoun as well as social-orientation words (i.e., words denoting social processes including all personal pronouns except for first-person singular pronouns and verbs referring to human interaction, e.g., *friends*, *talk*, or *share*), reflecting how often death row inmates referred to other people in their statements. Also, we determined LIWC results for cognitive-processing words (e.g., *think*, *know*, or *because*), indicating the extent to which death row inmates were concerned about intellectually understanding the topics addressed in their final statements, as well as past-tense, present-tense, and future-tense verbs as indicators of time orientation and words representing the linguistic categories of religion and death (cf. Pennebaker et al., 2007b; see also Cohn et al., 2004).

## Results

### Descriptive Results of Emotional Language Use in Executed Death Row Inmates’ Statements

**Table 2** presents the means and standard deviations of the LIWC output variables total number of words, and the percentages of categorized dictionary words, positive emotion words, and negative emotion words for spoken statements made by 407 executed death row inmates. The death row inmates’ statements contained a total of 42,328 words and on average 104 words per person. Of all words, an average of 92% of the words were categorized by the internal LIWC dictionary (see **Table 2**). A paired *t*-test indicated that, as predicted, executed death row inmates used on average a significantly higher proportion of positive emotion words (*M* = 9.64) than negative emotion words (*M* = 2.65), *t*(406) = 15.95, *p* < 0.001, *d* = 0.79.

**Table 2:** Descriptive results of LIWC analyses for spoken statements by death row inmates executed between 1982 and 2015 (June 30) in Texas.

	All spoken statements				Spoken statements with ≥3 words categorized by LIWC’s internal dictionary				Spoken statements with ≥10 words categorized by LIWC’s internal dictionary				Spoken statements controlled for outliers (*M* ± 3 *SD*)			
				
Total authors	407				403				381				385			
Total words	42,328				42,320				42,154				38,162			
				
LIWC variables	*M*	*SD*	Min	Max	*M*	*SD*	Min	Max	*M*	*SD*	Min	Max	*M*	*SD*	Min	Max
Word count	104.00	109.60	1.0	1268.0	105.01	109.67	3.0	1268.0	110.64	110.19	11.0	1268.0	99.12	80.38	3.0	405.0
Dictionary words (%)	92.43	8.91	0	100.0	92.93	5.97	59.4	100.0	93.26	5.25	59.4	100.0	93.05	5.55	66.7	100.0
Positive emotion words (%)	9.64	7.66	0	100.0	9.40	6.08	0	50.0	9.01	5.08	0	33.3	9.14	5.21	0	31.8
Negative emotion words (%)	2.65	3.08	0	33.3	2.67	3.08	0	33.3	2.74	2.68	0	20.8	2.52	2.40	0	10.8


On the sample level, to account for the different number of total words spoken by death row inmates, we computed the absolute numbers of positive emotion words and negative emotion words. Death row inmates’ use of total positive emotion words ranged from 0 to 50 (*M* = 7.94, *SD* = 6.88) words, and word use for negative emotion words ranged from 0 to 27 (*M* = 2.88, *SD* = 3.54) words. In order to analyze whether facing one’s own imminent death influenced positive emotional language use above and beyond the use of negative emotion words on an individual level, we calculated a positivity score for each death row inmate by subtracting the total number of negative emotion words from the total number of positive emotion words. This positivity index, ranging from -15 to 31, was greater than zero for 335 death row inmates (82.3%). The averaged positivity score was *M* = 5.07 (*SD* = 5.91) and differed significantly from zero, *t*(406) = 17.30, *p* < 0.001, *d* = 0.86.

Moreover, to demonstrate the robustness of the LIWC results for emotional language use in this unique sample of executed death row inmates, **Table 2** also provides the means and standard deviations of the LIWC output variables only for statements containing a minimum of three words that were categorized by LIWC’s internal dictionary and a minimum of 10 categorized words, respectively. Further, to control for effects of outliers, we analyzed the relevant LIWC output variables excluding the death row inmates’ statements that differed more than 3 *SD* from the means reported for the LIWC variables word count, dictionary words, positive emotion words, and negative emotion words (see **Table 2**). In all analyses, the LIWC results for positive emotional language hardly differed at all and accounted on average for at least 9% of the spoken words. Because the LIWC results proved to be very robust, the following comparisons with word usage base rates as well as word use preceding contemplated, attempted, and actual death were computed for the total sample of *N* = 407 death row inmates for whom transcribed spoken last statements were available.

### Comparison of Positive Emotional Language Use in Executed Death Row Inmates’ Statements with Word Usage Base Rates

**Table 3** lists descriptive results for the LIWC variable positive emotion words reported by Pennebaker et al. (2007b) from analyses of the written texts or speeches of *N* = 23,173 individuals—across a wide range of ages and social classes and including elementary-aged individuals, college students, elderly participants, and also prisoners—totaling over 168 million words. These results—spanning various contexts including science articles, novels, blogs, emotional writing (writing about deeply emotional topics), control writing (writing about trivial topics, such as plans for the day), and talking—provide an estimate of word usage base rates and reflect the degree to which different linguistic categories are found in various contexts. Across this large array of analyzed texts and speeches, positive emotional language use averaged to a grand mean of 2.74 (grand *SD* = 1.27) across all contexts, ranging from *M* = 1.33 for science articles to *M* = 3.72 for blogs and including *M* = 3.28 for emotional writing and *M* = 3.42 for talking (cf. Pennebaker et al., 2007b, p. 11). We computed independent *t*-tests to compare positive emotion word use in statements of executed death row inmates with the word usage base rates for positive emotions across all contexts as well as specifically for emotional writing and talking, respectively. As predicted, the results indicated that last statements spoken by executed death row inmates contained a significantly higher proportion of positive emotion words compared with base rates of positive emotional word usage analyzed by Pennebaker et al. (2007b), all *p*s < 0.001, *d*s > 1.1 (see **Table 3**). In addition, we used independent *t*-tests to analyze whether the percentage of negative emotion words spoken by *N* = 407 death row inmates in the final moments prior to their execution differed from word usage base rates. Death row inmates’ negative emotion word use (*M* = 2.65, *SD* = 3.08; see **Table 2**) was significantly higher than the word usage base rates across all contexts (grand *M* = 1.63, grand *SD* = 0.91), *t*(23,578) = 20.64, *p* < 0.001, *d* = 0.45, as well as talking (*M* = 1.49, *SD ≈* 0.91), *t*(1,255) = 10.10, *p* < 0.001, *d* = 0.51, but did not differ significantly compared with the word usage base rates from emotional writing (*M* = 2.67; *SD ≈* 0.91), *t*(1,419) = -0.19, *p* = 0.851, *d* = -0.01 (cf. Pennebaker et al., 2007b, p. 11).

**Table 3:** Comparison of positive emotional language use in executed death row inmates’ statements (*N* = 407, *M* = 9.64, *SD* = 7.66) with word usage base rates (Pennebaker et al., 2007b) and language use preceding contemplated death (Kashdan et al., 2014) and attempted or actual death by suicide (Handelman and Lester, 2007).

	Total authors	Total word count	Positive emotion words (%)		Independent *t*-test		
					
			*M*	*SD*	*t*	*df*	*p*	Cohen’s *d*
Pennebaker et al., 2007b								
Across all contexts	23,173	168,345,504	2.74	1.27	85.66	23,578	<0.001	1.26
Emotional writing^a^	1,014	1,299,400	3.28	n/a (≈1.27)	25.59	1,419	<0.001	1.16
Talking^a^	850	1,202,015	3.42	n/a (≈1.27)	23.03	1,255	<0.001	1.13
Kashdan et al., 2014								
Experiment 1A: MS condition^b^	n/a (≈136)	n/a	3.67	3.78	8.74	541	<0.001	0.99
Experiment 1B: MS condition^b^	n/a (≈43)	n/a	3.84	2.97	4.92	448	<0.001	1.00
Handelman and Lester, 2007								
Suicide notes: attempters^c^	20	n/a	3.55	n/a (≈7.66)	3.47	425	<0.001	0.80
Suicide notes: completers^c^	20	n/a	5.32	n/a (≈7.66)	2.46	425	0.014	0.56


*n/a, Information not available in the original study; MS, mortality salience. All *p*-values are two-tailed.*

*^a^As specific standard deviations for emotional writing and talking were not available, independent t-tests were computed with the grand SD = 1.27. ^b^As exact sample sizes for the MS conditions were not available, independent t-tests were computed with approximated sample sizes calculated by dividing the overall sample size of Experiments 1A,B by the number of conditions, respectively. ^c^Means for Handelman and Lester (2007) are weighted means calculated across sexes within groups of suicide attempters and suicide completers, respectively. As standard deviations for positive emotion word use were not available, independent t-tests were computed using SD = 7.66 of executed death row inmates as a conservative approximation.*

### Comparison of Positive Emotional Language Use in Executed Death Row Inmates’ Statements with Word Usage Preceding Contemplated, Attempted, and Actual Death

**Table 3** also presents results for the LIWC variable positive emotion words from recent research by Kashdan et al. (2014) examining effects of contemplated death on positive emotional language use in written narratives. We calculated independent *t*-tests to compare results for the mortality salience conditions in Kashdan et al.’s (2014) Experiments 1A,B with the statements of the executed death row inmates. The age and gender of participants in Kashdan et al.’s (2014) Experiment 1A were not recorded, but the undergraduate sample was taken from introductory psychology classes that were 70% female and consisted of students who were approximately 19 years old. In Experiment 1B, participants’ average age was 18.73 years, and 53.5% were female (cf. Kashdan et al., 2014, p. 172). We divided the overall sample size by number of conditions and used the approximated sample sizes of *n* = 136 (Experiment 1A) and *n* = 43 (Experiment 1B), respectively (as the precise sample sizes per condition were not available in the paper by Kashdan et al., 2014). As predicted, the results indicated that executed death row inmates used a significantly higher percentage of positive emotion words in the statements they made minutes before their own deaths compared with individuals contemplating their own deaths, all *p*s < 0.001, *d*s > 0.9 (see **Table 3**).

Next, we compared the proportion of positive emotional content in suicide notes preceding attempted or actual death reported by Handelman and Lester (2007) with positive word use by executed death row inmates using independent *t*-tests. Suicide notes composed before attempted suicides were written by individuals (seven males, 13 females) with an age range of 13–70 (Mdn = 32) years. Suicide notes composed before completed suicides were written by individuals (12 males, eight females) with an age range of 19–67 (Mdn = 35) years (cf. Handelman and Lester, 2007, p. 103). Means of positive emotion word use for the study by Handelman and Lester (2007) presented in **Table 3** are weighted means calculated across sexes within groups of attempted and completed suicides, respectively. We used the standard deviation of the LIWC variable positive emotion of Texas death row inmates (cf. **Table 2**, *SD* = 7.66) as a conservative approximated measure for the samples of attempted and completed suicides by Handelman and Lester (2007) as this information was not provided in the article. The results indicated that death row inmates used a significantly higher proportion of positive emotion words prior to their execution compared with individuals’ language use preceding attempted and actual death by suicide, all *p*s < 0.02, *d*s > 0.5.

With regard to psychological differences and similarities between suicide and execution, the last words spoken by a subgroup of death row inmates (i.e., so-called “volunteers”) who sought their own execution by abandoning their appeals could be considered (Blume, 2005; Vandiver et al., 2008). However, it remains unclear whether these consensual executions are attributable to the acceptance of responsibility and justness of the punishment or reflect the person’s seeking the aid of the state to commit suicide (cf. Vandiver et al., 2008, p. 940). In the analyzed sample of 407 death row inmates, the proportion of positive emotion words in the final statements of the 16 death row inmates who are listed as having volunteered for execution on the Death Penalty Information Center (2015b) website (*M* = 8.55, *SD* = 3.48) did not differ from the other 391 executions (*M* = 9.68, *SD* = 7.78), *t*(405) = -0.58, *p* = 0.561, *d* = 0.19.

### Additional Analyses

Further, we analyzed whether the use of positive emotional language in executed death row inmates’ final statements would be associated with language use indicative of self-references, social orientation, cognitive processing, time orientation, and personal concerns with religion and death. We computed correlations between the proportion of positive emotion words that were used and the respective LIWC variables, omitting two individuals with 0% categorized dictionary words from the correlation analyses. The descriptive and correlation results are presented in **Table 4.** As hypothesized, death row inmates’ positive emotional language use was associated with a greater use of social-orientation words, including words that refer to friends and present-tense verbs, all *p*s < 0.001. The findings also showed that emotional positivity in final statements was related to a more frequent use of first-person singular self-references as well as to less use of cognitive-processing words, past-tense verbs, and death-related words, all *p*s < 0.010.

**Table 4:** Descriptive statistics for language use indicative of self-references, social orientation, cognitive processing, time orientation, and personal concerns with religion and death and associations with positive emotional language use in executed death row inmates’ statements.

	Descriptive statistics		Positive emotion words (%)	
		
LIWC variables (in %)	*M*	*SD*	*r*	*p*
First-person singular pronouns	13.34	5.51	**0.14**	0.006
Social processes	17.67	8.57	**0.30**	<0.001
Family	1.99	2.41	0.02	0.683
Friends	0.33	1.10	**0.22**	<0.001
Humans	1.08	1.59	-0.02	0.706
Cognitive processes	15.42	6.32	**-0.28**	<0.001
Past tense	2.42	2.49	**-0.31**	<0.001
Present tense	15.68	7.33	**0.19**	<0.001
Future tense	1.66	2.48	**-**0.07	0.169
Religion	2.89	4.54	0.07	0.146
Death	0.70	1.48	**-0.17**	<0.001


**N* = 405. Significant correlations are printed in bold.*

### Supplementary Analyses

In addition, supplementary analyses (see **Supplementary Figure S1** and **Supplementary Tables S1** and **S2**, available online) showed that the findings of strong emotional positivity in death row inmates’ final statements were very robust with regard to demographic variables. We computed correlations between death row inmates’ positive word usage and the demographic variables age at execution, age at incarceration, years on death row, and educational level (i.e., highest grade completed), omitting two individuals with 0% categorized dictionary words from the correlation analyses. The proportion of positive emotional language use in the sample of 405 death row inmates was not significantly correlated with age at execution, *r*(403) = -0.04, *p* = 0.469, age at incarceration, *r*(387) = -0.03, *p* = 0.586, years spent on death row, *r*(387) = -0.04, *p* = 0.469, or educational level, *r*(377) = -0.05, *p* = 0.307. Moreover, neither the percentage of negative emotion words used nor the calculated positivity index was significantly correlated with the four demographic variables that we considered, all *r*s < | 0.08|, all *p*s > 0.150. Comparing the proportion of death row inmates’ positive emotional language use across ethnic backgrounds revealed significant differences, *F*(3, 403) = 5.20, *p* = 0.002 (see **Supplementary Table S1** available online). *Post hoc* tests (Tukey’s HSD) showed that White death row inmates used significantly fewer positive emotion words compared with Black death row inmates (*p* = 0.001), but despite these ethnic differences, the percentage of positive emotion words used by White death row inmates (*M* = 7.99) was still significantly higher than found in the suicide notes of completed suicides (Handelman and Lester, 2007), *t*(196) = 2.09, *p* = 0.038, *d* = 0.41. Additional analyses showed no significant differences in ethnic background for the percentage of negative emotion words used, *F*(3, 403) = 0.15, *p* = 0.931, and the calculated positivity index, *F*(3, 403) = 1.48, *p* = 0.220. Further, with regard to situational characteristics, the percentage of positive emotion words used did not change significantly from before to after the Texas Board of Criminal Justice allowed victim witnesses to attend executions in January 1996 (Texas Department of Criminal Justice, 2014), *p* = 0.222, *d* = 0.22 (see **Supplementary Table S2** available online). The percentage of negative emotion words used was slightly higher after victims’ families and individuals close to victims were permitted to attend executions, *p* = 0.040, *d* = -0.30, but as the average absolute number of words spoken by death row inmates was higher after this change, *p* < 0.001, *d* = -0.79, the positivity index was significantly higher after victim witnesses were allowed to attend executions (*M* = 5.50, *SD* = 6.15) than before (*M* = 2.20, *SD* = 2.62), *t*(405) = -3.89, *p* < 0.001, *d* = -0.70 (see **Supplementary Table S2** available online).

## Discussion

In the current study, using a computerized quantitative text analysis approach in a unique sample of Texas death row inmates executed between 1982 and 2015, we were able to show that the salience of one’s own imminent mortality was reflected in the emotional positivity of spoken final statements. That is, on the sample level, we were able to demonstrate that death row inmates used a significantly higher percentage of positive emotion words than negative emotion words in their final statements. Moreover, on the individual level, a positivity index, which was statistically different from zero on average, showed that statements by over 80% of executed death row inmates contained more positive than negative emotion words.

More importantly, our findings are the first to demonstrate that executed death row inmates use a significantly higher percentage of positive emotion words compared with base rates of word usage from heterogeneous samples and various contexts (cf. Pennebaker et al., 2007b). In addition, we were able to show that executed death row inmates’ statements contained a significantly higher proportion of positive emotion words than the writings of individuals contemplating death (cf. Kashdan et al., 2014) as well as compared with writings preceding attempted or actual suicide (cf. Handelman and Lester, 2007). Due to the large sample size and the various control analyses, our results on emotional positivity can be viewed as robust. In sum, the final statements of Texas death row inmates conveyed extremely positive expressions that reflected the emotional processes of coping with mortality. In addition, emotional positivity in spoken final statements was shown to be associated with a greater frequency of language use indicative of self-references, social orientation, and present-oriented time focus as well as with fewer instances of cognitive-processing, past-oriented, and death-related word use.

In line with TMT’s proposed psychological defenses that are aimed at avoiding or ignoring the anxiety that mortality salience evokes (Pyszczynski et al., 1999), our findings contribute to the burgeoning literature that suggests elevations in positive emotional language use as an immediate way of coping with the immense threat of one’s imminent death (see DeWall and Baumeister, 2007; Kashdan et al., 2014). The presence of such a large amount of emotional positivity despite facing one’s actual death raises interesting psychological issues and seems clearly counterintuitive. In the fully constrained moments before execution while undergoing standardized execution procedures and being strapped to a gurney (Texas Department of Criminal Justice, 2012), death row inmates have little control over their situation with the exception of the opportunity to make a final statement. To defend against death anxiety, death row inmates seem to show an intense impulse to embrace emotional positivity. This might reflect underlying motivational mechanisms by which people value meaningful close others and focus on the moment rather than on the past or distant future, and it might originate from the perception of time limitations on life itself as postulated by SST (Carstensen et al., 1999). Thus, the public policy of allowing the death row inmate a final opportunity to speak to family and friends, those close to the victim(s), prison officials, as well as the general public may be viewed as one act through which death row inmates may linguistically regulate their intense emotions and experience some form of perceived control in the final moments of life (see also Vollum and Longmire, 2009; Ward, 2010). However, larger ethical, moral, and legal issues of capital punishment still remain (e.g., Bohm, 2008; for a database of exonerated, innocent death row inmates, see the Death Penalty Information Center, 2015a website).

Despite the robust findings of a tuning in to emotional positivity in death row inmates’ last statements reflected by a quantitative text analysis, several limitations need to be considered. First, we were not able to examine the defensive psychological mechanisms of death salience for nearly 23% of the death row inmates—those who chose to remain silent. Nevertheless, demographic comparisons provided evidence that these individuals did not differ significantly from the death row inmates who made final statements. Second, due to a lack of more detailed information on each individual death row inmate, contextual factors caused by death row confinement, behaviors prior to execution, and situational factors in the death chamber were not comprehensively taken into account. We could not rule out the possibility that the conditions of life on death row (i.e., solitary confinement while awaiting death usually for years) and its psychological consequences (see e.g., Johnson, 1979; Cunningham and Vigen, 2002) may have influenced the delivery of last words. Also, behaviors prior to execution (e.g., an apologetic confession to the offense) were not considered in the analyses of final words. According to prior research on the positive consequences of emotional disclosure (Pennebaker, 2003), it is possible that a positive psychological impact could have occurred for death row inmates who had confessed at some time prior to execution (Umbreit and Vos, 2000). Further, despite the extremely standardized execution protocol, the possible influence of situational characteristics (e.g., the actual presence or absence of one’s own loved ones and victim witnesses) during the execution could not be examined.

Future research may investigate other forms of communication by death row inmates (e.g., diaries or letters written before death) to provide further empirical support for the emotional tuning in to positivity as a coping mechanism for regulating anxiety about one’s mortality. Also, future research could analyze language use in other samples facing death, such as terminally ill individuals (e.g., hospice patients). Moreover, people’s last words before death published on online social network sites such as Facebook or Twitter (e.g., Gunn and Lester, 2012) can provide researchers with further valuable insights into how people cope with death anxiety linguistically. More broadly, researchers, taking an appreciative approach to death (cf. Frias et al., 2011; Vail et al., 2012), should continue to examine the positive feelings and positive experiences that a person’s own imminent death can promote.

Taken together, in line with TMT’s postulation of psychological defenses against death anxiety (Pyszczynski et al., 1999), the present work demonstrates that a pronounced tuning in to emotional positivity manifests in final language use immediately before execution. Thus, our findings also support the notion that there is more psychological terror associated with being executed compared with (completed and attempted) suicide and contemplated death given that the tuning in to emotional positivity acts as a terror management mechanism. Considering real deaths by both execution and suicide, it remains an important task to further investigate why individuals facing execution use an even greater number of positive emotion words. Psychologists, taking account of the perspectives of death row inmates, victims, and society as a whole, should continue to shed light on individuals’ immediate coping with human mortality reflected in their words before death.

## Conflict of Interest Statement

The authors declare that the research was conducted in the absence of any commercial or financial relationships that could be construed as a potential conflict of interest.
