# Supplementary material for: Positive Emotional Language in the Final Words Spoken Directly Before Execution
Source: Front Psychol. 2016 Jan 13;6:1985. doi: 10.3389/fpsyg.2015.01985 (PMC4710806; doi:10.3389/fpsyg.2015.01985)
Supplement: Supplementary file 1 [file Supplementary_Material.PDF]

Supplementary Material

Positive Emotional Language in the Final Words Spoken Directly Before Execution

Sarah Hirschmüller*, Boris Egloff

*** Correspondence:** Sarah Hirschmüller: hirschmu@uni-mainz.de

1 Supplementary Figure and Tables

Fig. 1a

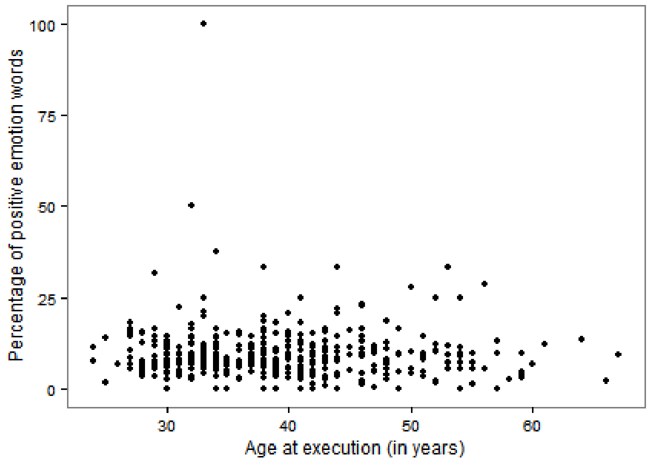

Fig. 1b

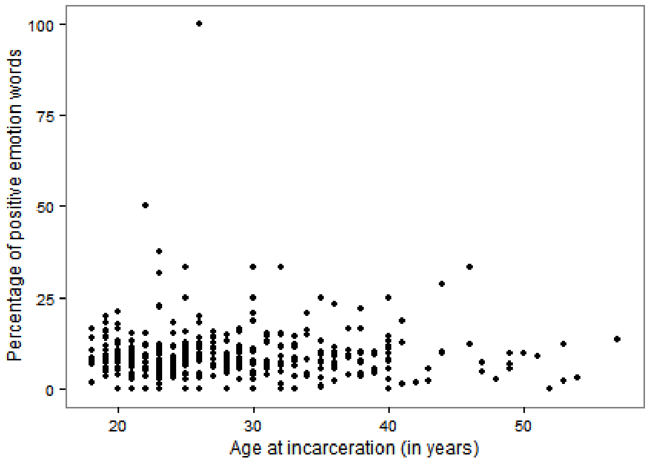

Fig. 1c

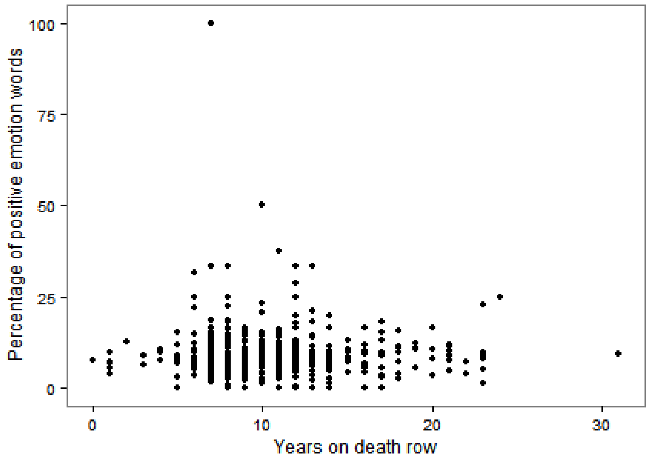

Fig. 1d

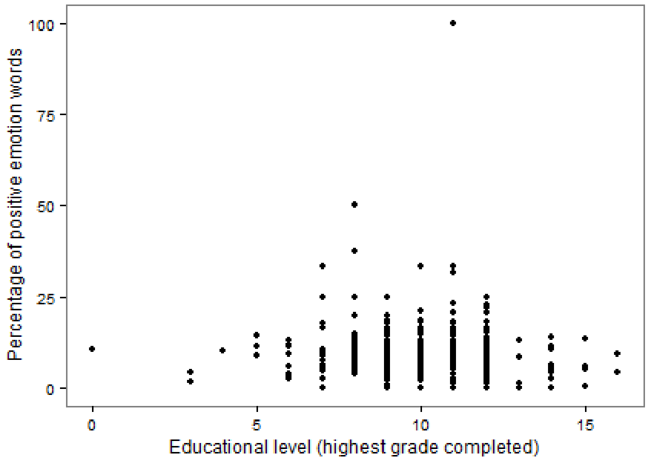

1.1 Supplementary Figure 1. Relations between Death Row Inmates' Percentage of Positive Emotion Word Use and the Demographic Variables Age at Execution (Fig. 1a), Age at Incarceration (Fig. 1b), Years on Death Row (Fig. 1c), and Educational Level (Fig. 1d).

1.2 Supplementary Table S1. Detailed Descriptive Results of LIWC Analyses of Statements Spoken by Death Row Inmates Organized by Ethnic Groups.

	**Whites**				**Blacks**				**Hispanics**				**Other**					
Total files	178				150				77				2					
Total words	19,050				15,482				7,695				101				*df*= 3, 403	
**LIWC variables**	***M***	***SD***	**Min**	**Max**	***M***	***SD***	**Min**	**Max**	***M***	***SD***	**Min**	**Max**	***M***	***SD***	**Min**	**Max**	***F***	***p***
Word count	107.02	99.84	1.0	688.0	103.21	129.77	1.0	1268.0	99.94	88.40	4.0	362.0	50.50	23.33	34.0	67.0	0.24	.868
Dictionary words (%)	91.65	11.56	0	100.0	93.13	6.24	59.4	100.0	92.71	5.74	70.0	100.0	97.76	3.17	95.5	100.0	1.03	.378
Positive emotion words (%)	7.99	5.12	0	33.3	11.13	10.29	0	100.0	10.56	5.72	0	31.8	8.89	0.10	8.8	9.0	5.20	.002
Negative emotion words (%)	2.72	2.56	0	12.5	2.59	2.90	0	20.8	2.62	4.33	0	33.3	1.50	2.11	0	3.0	0.15	.931
Positivity index	4.55	5.94	-14.0	29.0	5.12	5.71	-15.0	31.0	6.21	6.19	-2.0	28.0	3.50	0.71	3.0	4.0	1.48	.220

Note. Post hoc tests (Tukey's HSD) showed that White death row inmates used significantly fewer positive emotion words than Black death row inmates (p = .001). All p-values are two-tailed.

1.3 Supplementary Table S2. Detailed Descriptive Results of LIWC Analyses of Statements Spoken by Death Row Inmates Executed between 1982 and 2015 (June 30) in Texas Before and After Victim Witnesses were Permitted to Attend.

	**Attendance of victim witnesses not allowed** (before January, 1996)				**Attendance of victim witnesses allowed** (after January, 1996)						
Total files	54				353						
Total words	2,357				39,971						
**LIWC variables**	***M***	***SD***	**Min**	**Max**	***M***	***SD***	**Min**	**Max**	***t***	***df***	***p***
Word count [a]	43.65	50.66	1.0	267.0	113.23	113.24	1.0	1268.0	-7.60	151.6	<.001
Dictionary words (%)	91.08	9.22	59.4	100.0	92.63	8.86	0	100.0	-1.20	405	.232
Positive emotion words (%) [a]	11.78	14.52	0	100.0	9.31	5.92	0	50.0	1.23	55.7	.222
Negative emotion words (%)	1.85	2.98	0	12.5	2.77	3.08	0	33.3	-2.06	405	.040
Positivity index [a]	2.20	2.62	-2.0	12.0	5.50	6.15	-15.0	31.0	-3.30	162.2	<.001

Note. In January 1996, the Texas Board of Criminal Justice adopted a rule that permitted victim witnesses to attend executions (Texas Department of Criminal Justice, 2014). All *p*-values are two-tailed.

[a] An independent *t* test with unequal variances was used.
